# Phasing quality assessment in a brown layer population through family- and population-based software

**DOI:** 10.1186/s12863-019-0759-3

**Published:** 2019-07-17

**Authors:** N. Frioni, D. Cavero, H. Simianer, M. Erbe

**Affiliations:** 1 Animal Breeding and Genetics Group, Department of Animal Sciences, Center for Integrated Breeding Research, University of Goettingen, Göttingen, Germany; 2 H & N International, Cuxhaven, Germany; 3 Bavarian State Research Centre for Agriculture, Institute for Animal Breeding, Grub, Germany

**Keywords:** Haplotype phasing, Poultry data, Phasing quality, Phasing software, Beagle, FImpute

## Abstract

**Background:**

Haplotype data contains more information than genotype data and provides possibilities such as imputing low frequency variants, inferring points of recombination, detecting recurrent mutations, mapping linkage disequilibrium (LD), studying selection signatures, estimating IBD probabilities, etc. In addition, haplotype structure is used to assess genetic diversity and expected accuracy in genomic selection programs. Nevertheless, the quality and efficiency of phasing has rarely been a subject of thorough study but was assessed mainly as a by-product in imputation quality studies. Moreover, phasing studies based on data of a poultry population are non-existent. The aim of this study was to evaluate the phasing quality of FImpute and Beagle, two of the most used phasing software.

**Results:**

We simulated ten replicated samples of a layer population comprising 888 individuals from a real SNP dataset of 580 k and a pedigree of 12 generations. Chromosomes analyzed were 1, 7 and 20. We measured the percentage of SNPs that were phased equally between true and phased haplotypes (Eqp), proportion of individuals completely correctly phased, number of incorrectly phased SNPs or Breakpoints (Bkp) and the length of inverted haplotype segments. Results were obtained for three different groups of individuals, with no parents or offspring genotyped in the dataset, with only one parent, and with both parents, respectively. The phasing was performed with Beagle (v3.3 and v4.1) and FImpute v2.2 (with and without pedigree). Eqp values ranged from 88 to 100%, with the best results from haplotypes phased with Beagle v4.1 and FImpute with pedigree information and at least one parent genotyped. FImpute haplotypes showed a higher number of Bkp than Beagle. As a consequence, switched haplotype segments were longer for Beagle than for FImpute.

**Conclusion:**

We concluded that for the dataset applied in this study Beagle v4.1 or FImpute with pedigree information and at least one parent genotyped in the data set were the best alternatives for obtaining high quality phased haplotypes.

**Electronic supplementary material:**

The online version of this article (10.1186/s12863-019-0759-3) contains supplementary material, which is available to authorized users.

## Background

Phasing is the process of inferring haplotypes from genotypes. Haplotype data contain more information than genotype data, as they make it possible to track single alleles or haplotype segments back in the pedigree. The applications of haplotype information cover many fields of research in genetics. In livestock, haplotype structure can be applied to improve the accuracy in genomic selection programs. Although, the quality and efficiency of phasing has scarcely been a subject of thorough study [1–6]. Phasing quality has mainly been assessed as a by-product in imputation quality studies [7–10]. Furthermore, phasing studies based on data of a poultry population are non-existent.

Haplotypes can be obtained by phasing genotypes in silico. Software available at the moment for this purpose can be roughly divided into two groups: family-based and population-based phasing strategies [9]. Population-based algorithms exploit the LD between close SNPs to model haplotype frequencies while the family-based ones use linkage between close relatives. At present, FImpute [8] and Beagle [11] are two of the most known and widely used software for haplotype phasing.

FImpute assumes that common haplotypes between closely related individuals are longer than the ones shared by more distant individuals. The first step of the program, given that pedigree information is available, is to scan all chromosomes in known parent-offspring pairs. Without pedigree information, parent-offspring pairs are identified by matching long shared haplotypes. Later, the program iterates the pedigree up and down in order to search for more haplotype matches by applying an overlapping sliding window approach (OSW) along chromosomes. The OSW changes the size (in each chromosomal sweep) in order to find smaller haplotypes, but also to keep phase consistency between haplotypes and to increase phasing accuracy. The haplotype information is collected in a library, which later is used for identifying haplotypes of high similarity (≥99%), inferring haplotypes for heterozygous genotypes and calculating haplotype frequencies. As the accuracy in long windows is quite high, these segments are used as anchors for phasing smaller windows. Hence, more phasing errors can be expected at the beginning and end of long haplotype segments [8].

**Fig. 1:**
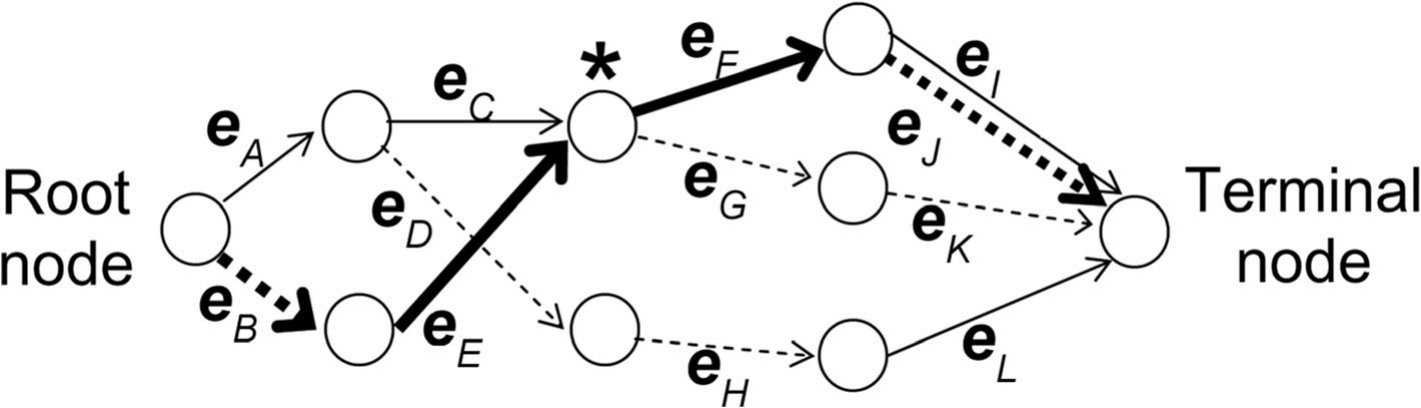
Example of a directed acyclic graph representing the localized haplotype-cluster model for four markers, adapted from Browning and Browning (2007)

The Beagle approach is based on a hidden Markov Model (HMM). The methodology is composed of two steps: *(i)* build a localized haplotype-cluster model (LHCM) based on LD between markers and *(ii)* execute the phasing program. The phasing execution is an iterative process which in each round *(a)* fits the LHCM to estimate haplotype information and *(b)* samples haplotypes conditional on the LHCM and genotyped data. The LHCM is an acyclic graph with a root and a terminal node connected by many intermediate nodes and edges. Each edge, being the connection between nodes, is a cluster of haplotypes. For example, the cluster of haplotypes for a given edge *e*_*i*_ may group all haplotypes whose path travels from the root node to the edge *e*_*i*_. Moving from one node to the next (one edge) towards the terminal node will increase the haplotypes size by one marker. Thus, a graph may have as many edges as haplotypes markers that are being modelled. Figure 1 adapted from [11] presents an example of such a graph. Beagle later iterates all the individuals using phased data as input and in each iteration samples diplotypes for each individual conditional to the respective individual genotype information. In Fig. 1, for each marker, allele 1 is represented by a solid line, and allele 2 by a dashed line. The bold line edges from the root node to the terminal node represent the haplotype 2112. This graph is an example of a HMM used by the phasing program.

A phasing quality study from Miar et al. (2017) tested (among others) FImpute v2.2 and Beagle v4.1 in phasing dairy cattle genotypes from low and high-density arrays. A high level of phasing accuracy (> 99%) was observed for both software, recommending FImpute as the faster option. Hickey et al. (2011) measured phasing quality for one chromosome of dairy and beef cattle, sheep, pig, and human populations with the software LRPHLI. In a comparison of different phasing strategies (computational and laboratory based) results of phasing quality were reported [13].The percentage of correctly phased alleles was above 97% for livestock and 93.7% for the human chromosome.

A recent study [14], included (among others) Beagle (v4.0) applied to human data (1 individual) with 1.6 million SNPs (on 22 autosome chromosomes) and used two reference panels: *(i)* 1000 Genome Project (1000GP) with 2.5 k individuals and *(ii)* Haplotype Reference Consortium (HRC) with 23 k individuals. The results obtained with Beagle presented 1.5% (1000GP as reference panel) and 0.5% (HRC as reference panel) wrongly phased alleles, respectively.

The aim of this study is to fill a gap in phasing quality studies, with three special features: *(i)* this study focuses only on phasing quality, *(ii)* it is based on real poultry data and *(iii)* uses simulated data with known haplotypes. We applied two software, FImpute v2.2 [8] with (FImpute) and without (FImpute np) pedigree information and Beagle in version v3.3 (Beagle 3) and v4.1 (Beagle 4) [11], to simulated genomic data based on a highly-related brown layer population.

## Results

### Equally phased SNPs

**Fig. 2:**
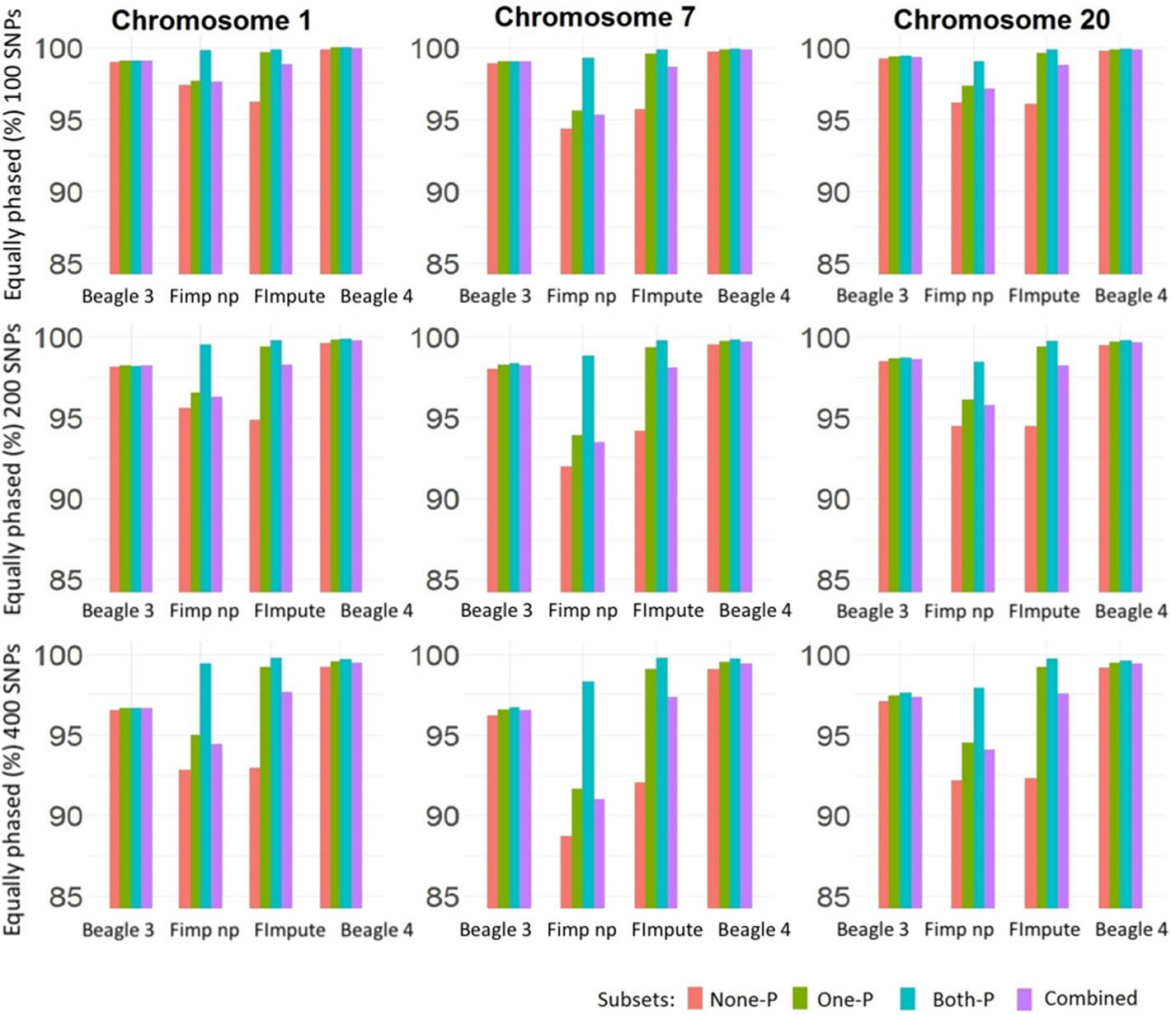
Equally phased values (%) for the subset None-P, One-P, Both-P and the combined subsets by chromosome (columns) and SNPs windows (rows) of analysis

This parameter was calculated for each individual’s haplotype within windows of 100, 200 and 400 heterozygous SNPs. Figure 2 presents median values from all the individuals and the 10 replicates by subsets and chromosomes.

The Eqp percentage values ranged from 88 to 100%. The exact values are presented in Additional file 1: Tables S1 to S3. The lowest value observed was for chromosome 7 (Fig. 2) phased with FImpute without pedigree information within the 400-window analysis for the None-P (individuals with no genotyped parents) subset. The highest value (99.99%) was obtained for chromosome 1 phased with Beagle v4.1 in the Both-P (individuals with both parents genotyped) subset. Regardless of the subset, haplotypes phased with Beagle v4.1 reached values above 99% in all scenarios.

Across the different chromosomes and/or windows the impact of genotyped relatives in the data set was as expected. The phasing quality increased when a given individual had one or both parents in the dataset. However, the improvement was different regarding the software used. With FImpute, the Eqp values increased when going from the None-P subset to Both-P. This pattern was more pronounced when the window size was 400 SNPs. Further, genotyped close relatives were found to have a lesser effect when phasing with Beagle than with FImpute.

Regardless of the subset, Eqp values obtained with FImpute were in general higher when obtained with pedigree information than those obtained without it. Moreover, results obtained with FImpute with pedigree information showed less variation across subsets (Fig. 2). With genotyped relatives in the dataset (One-P and Both-P) Eqp values of FImpute with pedigree information were very similar ranging from 99.1 to 99.8% while Eqp values of FImpute without pedigree information ranged between 91.7 and 99.8% (Additional file 1: Table S1 to S3). The highest Eqp values with FImpute were observed with pedigree information and both parents genotyped, reaching values similar to Beagle v4.1.

Beagle phased haplotypes exhibited different features than those phased with FImpute. Eqp values reached with version v3.3 were lower than with version v4.1. Only when increasing the window size in the analysis the Eqp values obtained with Beagle v3.3 became lower, at a similar rate for each subset and regardless the chromosome size.

**Fig. 3:**
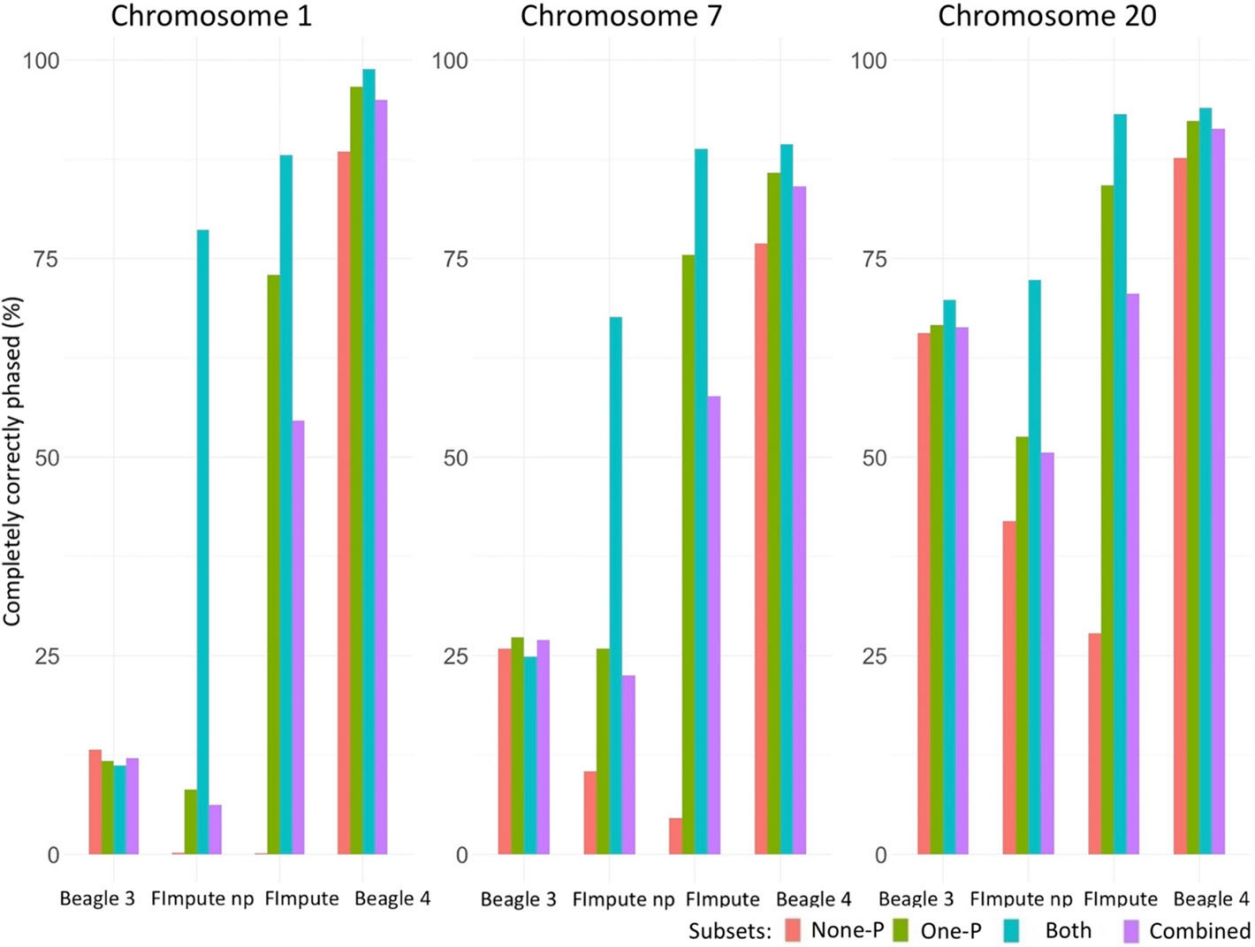
Proportion (%) of completely correctly phased individuals for chromosomes 1, 7 and 20 (columns) by subsets

In Fig. 3, the number of individuals which were completely correctly phased is shown. In this parameter the effect of genotyped relatives in the sample was bigger for FImpute than for Beagle. Beagle v3.3 showed a significant effect of chromosome size. While for chromosome 1 the 100% correctly phased individuals did not reach 25%, this value exceeded 60% in chromosome 20. While one would expect that it should be easier to phase a short chromosome entirely correctly compared to a long chromosome, this pattern is not found both with Beagle v.4.1 and FImpute with pedigree information when both parents were observed.

### Breakpoints

**Fig. 4:**
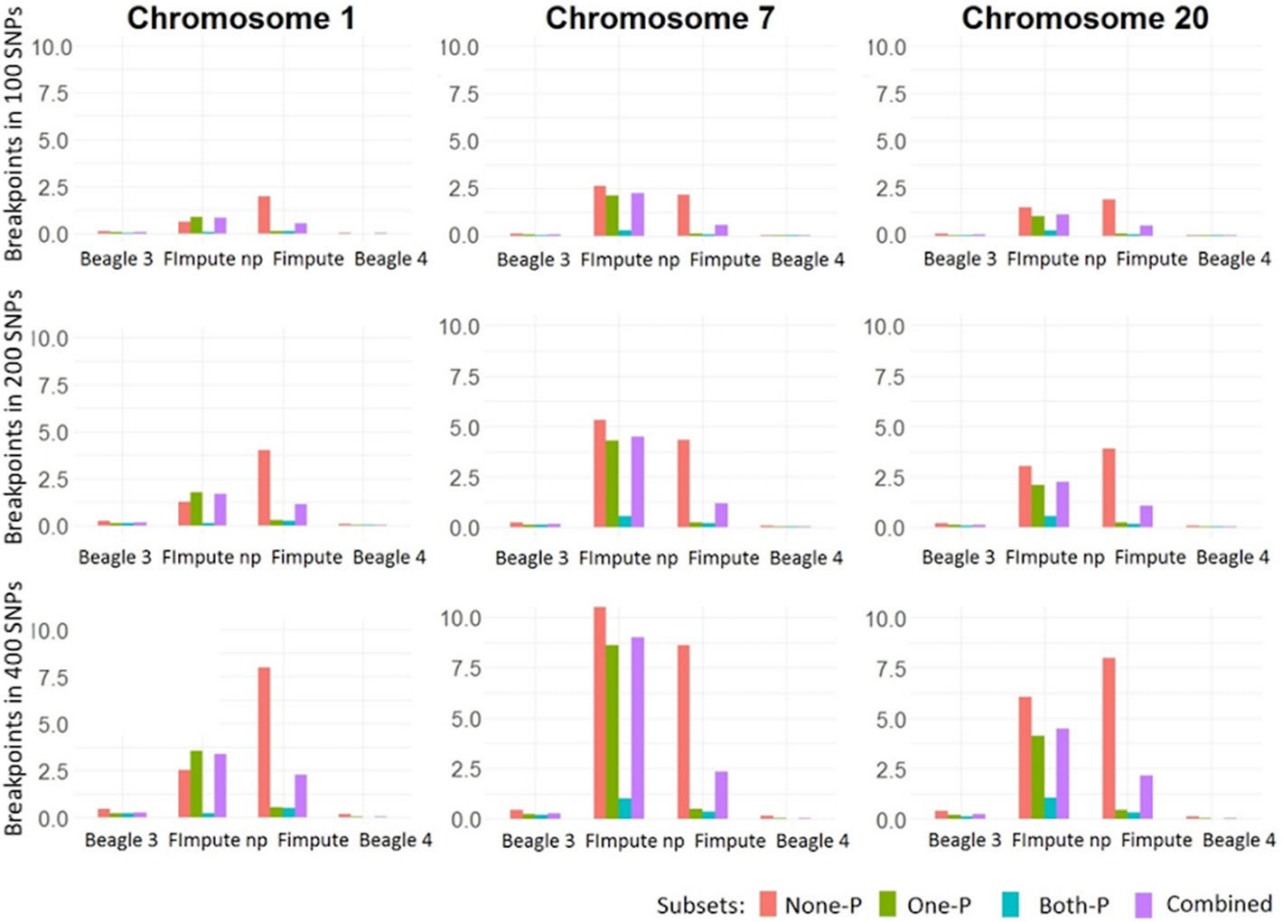
Breakpoint values for the subset None-P, One-P, Both-P and the combined subsets by chromosome (columns) and SNPs windows (rows) of analysis

The median values for Bkp by chromosomes and subsets for all individuals and replicates are shown in Fig. 4. The number of breakpoints showed an increasing pattern when enlarging the SNPs window size. Moreover, the Bkp values exhibited a “profile” regarding the software used. While with Beagle (either version) the median values of Bkp remained below 1 regardless of window size, chromosome or subset, Bkp values were higher with FImpute in many scenarios. However, FImpute showed a different behavior across subsets. When phased individuals did not have genotyped parents (None-P) the value of Bkp was higher than when individuals had genotyped parents (One-P and Both-P).

Regarding the different approaches of phasing with FImpute, haplotypes phased with pedigree information showed in general a lower amount of Bkp compared to haplotypes phased without pedigree information. With absence of pedigree information results for the None-P and One-P subsets presented similar and much higher Bkp values than for the Both-P subset. When pedigree was available the results for the One-P and Both-P subsets were rather similar with a very low number of Bkp, while the None-P subset showed higher values.

### Switched haplotype segments

**Fig. 5:**
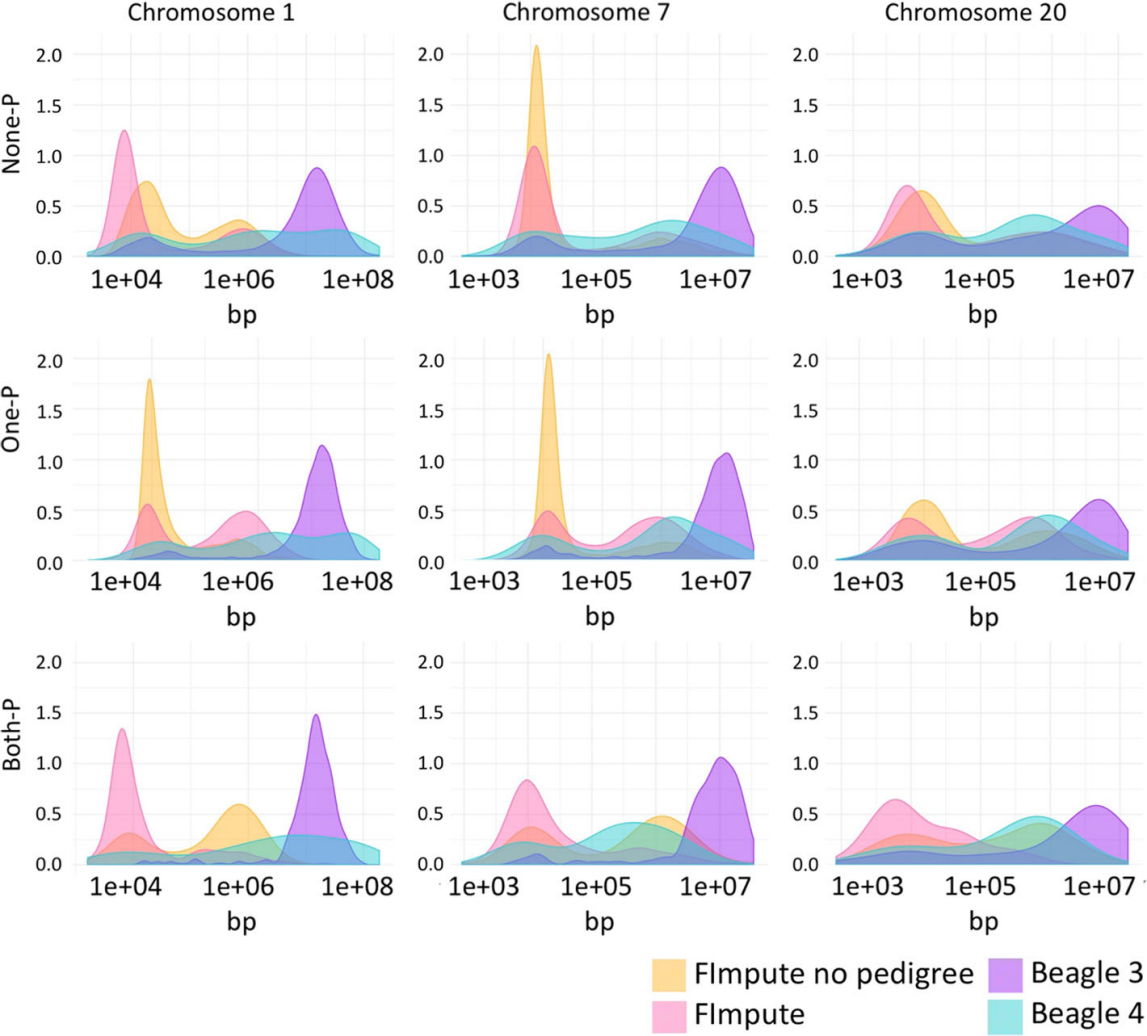
Switched haplotype segment size for each software by subset (rows) and by chromosome (columns)

These segments were not measured within windows like the previous quality parameters (Eqp and Bkp) but chromosome wide. Length was measured from the middle position between a correctly and incorrectly phased SNP for both start and end points. In Fig. 5 the logarithm of the distances is presented in density plots for each chromosome and subset.

FImpute peaks were in general placed on the left, representing shorter segments, while at the end of the axis there was always a blue peak corresponding to Beagle v3.3. This indicates a different profile in the switched segment size of these two software.

If analyzed within chromosomes and across the different subsets only FImpute shows a change of the shape of the curves. When haplotypes were phased without pedigree information in chromosome 1 going from None-P to One-P increased the amount of short switched segments while the amount of medium sizes segments was moderately reduced. For this chromosome in the Both-P subset the amount of short switched segment became much less and there was an increment in the medium size ones. This behavior was observed to be quite similar for the other chromosomes analyzed.

For FImpute with pedigree information, going across subsets presented a different behavior. When going from None-P to One-P the number of short segments became less while at the same time medium size segments slightly increased. In the Both-P subset the number of short segments increased to a similar level as in the None-P subset while the number of medium size segments reached the lowest level. For the other chromosomes the curves’ shape showed similar changes across subsets.

Beagle v3.3 results did not change substantially across subsets but across chromosomes. Haplotypes switched segments phased with v3.3 showed a decrease when moving from big size chromosome to shorter ones. With version v4.1 there was no clear trend neither across subsets nor chromosomes. These haplotypes presented a quite constant profile in switched segments, being in general accumulated in medium-long sizes. The values for different chromosomes by subset and software are presented in Additional file 1: Table S1-S3. FImpute with pedigree information, as well as Beagle v3.3, showed a pattern across chromosomes. When moving to smaller chromosomes there was a clear trend to a lower number of switched segments.

### Progeny effect

In addition, to understand if progeny had a relevant effect on phasing quality we grouped the individuals by the amount of progeny, the results are displayed in Additional file 1: Table S7. We calculated mean values of Eqp and Bkp for individuals with 0, 1 or 2, and more than 2 progenies. FImpute with pedigree and Beagle v4.1 did not show substantial differences of Eqp between these groups. However, Bkp values of FImpute phased haplotypes (with and without pedigree information) decreased when more progeny was available in the dataset. Beagle 3.3 also showed a similar decreasing Bkp pattern, but with absolute values lower than FImpute.

## Discussion

### Simulated data

For this study the known (true) haplotypes were obtained through a simulation. For this process, real SNP data was used as the main input and the homozygosity levels were used as a reference to produce haplotypes similar to reality. The homozygosity observed in real data was 72% for chromosome 1 and 7 and 74% for chromosome 20. The simulated data presented on average 67% for chromosome 1 and 7 and 68% for chromosome 20.

**Fig. 6:**
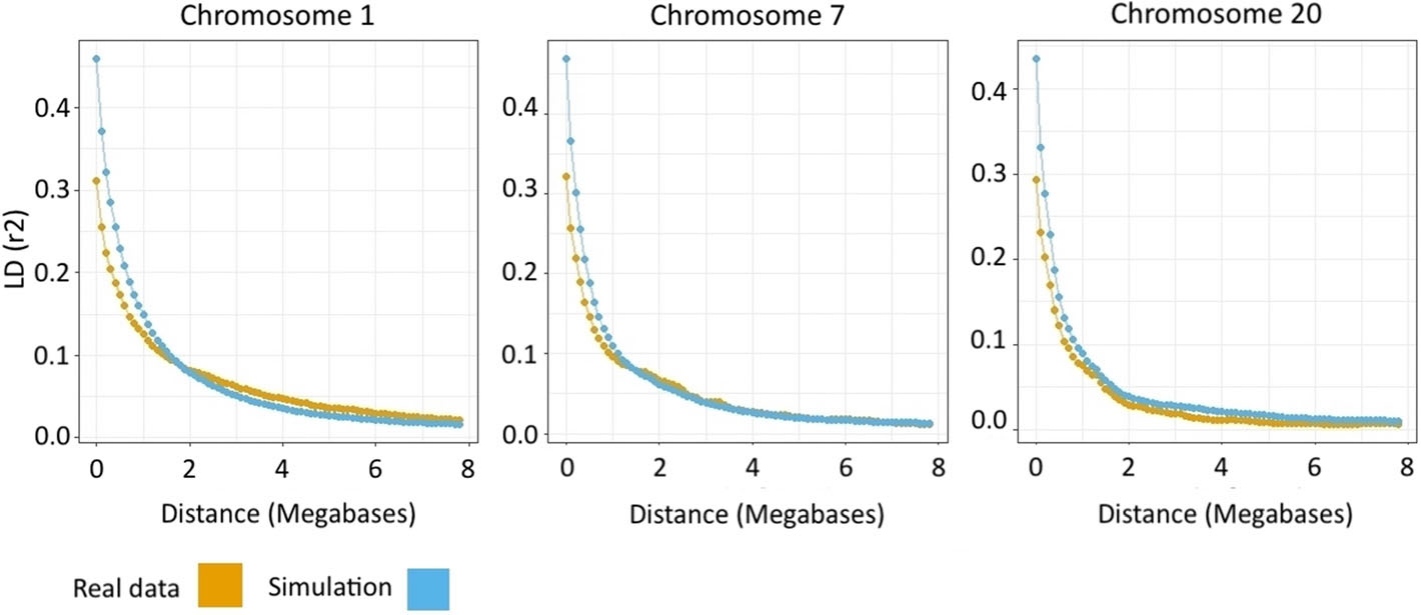
LD block density for real and simulated data

In Fig. 6 the LD decay of real and simulated data, as an example of the quality achieved, is presented for each chromosome. The mean LD values decayed while increasing the pairwise distance, though simulated data exhibited slightly higher values of LD at shorter distance than the real data. The simulation performed in this study allows us to evaluate the phasing software with in silico created haplotypes that are representative of real haplotypes.

### Phasing quality

Beagle v4.1 stood out presenting the steadiest and highest results for each chromosome, subset or window of analysis. However, FImpute with pedigree information and at least one parent genotyped achieved similar results. Our findings are in agreement with others previously reported [6, 12–14]. Although the consulted literature may differ in datasets and evaluated software, FImpute and Beagle phased haplotypes have consistently stood out as the best alternatives [6, 12, 14]. Beagle v3.3 was reported as the best option when evaluated with human data [6] and Wellcome Trust Case Control Consortium 2 data [15] and compared with Impute 2.1 [7] and Mach 1.0 [16] for accuracy and computing time. Another study [12] reported FImpute 2.2 to be the best alternative, compared to Findhap 3 [17], Beagle 4.1 and ShapeIt2 2.12 [18] with a dairy cattle dataset.

As observed in the current study and by Miar et al. [12], information from relatives provided a leap forward in the quality of the haplotypes obtained with FImpute. An alternative to closely related individuals is to use a reference panel. This is a more realistic resource in human genetics, as exploited by Choi et al. [14], but not in the standard case of livestock research. In this study we did not have a reference panel, but we studied the impact of information from relatives by grouping the individuals in three subsets for the main analyses: None-P, One-P and Both-P.

The Bkp values, the number of times within a window a change of phased occurred, were very different between FImpute and Beagle haplotypes. This reflected the algorithms behind the software, which followed different approaches. Across subsets, the highest difference of Bkp was observed for Chromosome 7, which ranged from 1 to 10, phased with FImpute without pedigree with window size 400, where None-P and Both-P Eqp values obtained were 88.7 and 98.3%, respectively. A similar behavior was observed by Miar et al. [19] measuring haplotype length accuracy (length of correctly phased haplotype segments) across datasets with no parents, pairs and trios with trios presenting the highest values. In the trios scenario it is easier for FImpute to find long shared haplotypes. These haplotypes are used as anchors and adjacent (shorter) haplotypes are later attached when the overlapping sliding window shrinks enough to find suitable (candidate) haplotypes, filling blank spaces, though probably with higher error probability resulting in Bkps. Beagle, following a different concept, creates a cluster of haplotypes which is improved through iterations on a HMM. Each individual’s haplotype is reconstructed due to genotype information and haplotype cluster probability.

Our results for Bkp increased with the size of window of analysis. Thus, we calculated the Bkp relative to the window size used (Additional file 1: Table S8) and observed no variation across window sizes. While applying longer windows of analysis we were covering more SNPs and increased the chance of finding switched SNPs.

The third quality parameter measured was the switched segment size, which can be seen as the interaction of both, the Eqp and Bkp values. FImpute without pedigree and parents genotyped registered the highest density of values for short segments. As mentioned above, short switched segments are created by the software, which can be a problem when studying either a specific region of a chromosome or few loci. Such a situation can be solved if genotyped parents can be added to the dataset in addition to a pedigree file. Haplotypes phased with Beagle v3.3 presented the highest density of long switched segments, these haplotypes had at the same time the lowest Eqp values. Beagle v4.1 presented a lower density of switched segments than v3.3 and did not exhibit a specific size profile but size segments were more uniformly distributed compared with the rest of the software evaluated.

### Computing time

The computing time was faster for FImpute than for Beagle. All runs were performed with default settings, meaning 10 jobs in parallel for FImpute and 10 iterations for Beagle. For chromosome 1 FImpute with pedigree information required on average 7 min 27 s, without pedigree information the time needed was 6 min and 26 s. With Beagle v3.3 running time was 8 h 24 min, while it was 3 h 26 min with Beagle v4.1, both versions operating with 15Gb of RAM.

### Missing values

Having data with missing values is a common situation in real data sets. Our results based on simulated haplotypes without missing values may differ from results of real data where a small proportion of genotypes is typically missing. Therefore, we created 3 replicates with 3% missing values, which was the typical level found in the real data after applying quality filters. The 3% SNPs were deleted randomly for each individual. This was repeated 5 times with different starting seeds to reduce the probability of deleting important SNPs by chance. Finally, we had 15 files per chromosome with 3% missing values each.

These files were analyzed exactly the same way as the as the ones without missing SNPs. The results varied only slightly from the results reported and can be found in the, Additional file 1: Table S4 to S6. It is concluded that missing values have no systematic effect on the reported pattern of phasing quality.

## Conclusions

For the poultry data analyzed, the best options for phasing were Beagle v4.1 and FImpute with pedigree information with at least one parent genotyped. The switched segments observed for the best two options (Beagle v4.1 and FImpute with pedigree information) do not compromise the overall quality of the reconstructed haplotypes. Since for most data sets there will be a certain proportion of individuals without genotyped parents and progeny, Beagle v4.1 appears to be the most robust and recommendable option when phasing quality is of interest, despite the fact that computing time is longer compared to FImpute.

## Methods

### Data and editing

In this study we used the pedigree information of 1′768 individuals of a purebred line of commercial brown layers. The pedigree contained information from 13 generations. The genomic data comprised information of 918 individuals from the pedigree that were members of generations 7 to 12. The genotyping array used was the Affymetrix Axiom® Genome-Wide Chicken Array with around 580 k SNPs [20].

The genomic data was edited with PLINK [21]. Individuals with a call rate < 90% were discarded. Monomorphic SNPs, SNPs not in autosomes and SNPs which were not in Hardy-Weinberg equilibrium with *p* < 10^− 8^ were removed. After editing, 888 genotyped individuals with 416 k autosomal SNPs remained in the dataset.

For practical reasons we analyzed one large, medium and small size chromosome, respectively, considered as being representative of the *Gallus gallus* genome (35 chromosomes in the reference genome). The selected ones were 1, 7 and 20 which after editing contained 77′910 (195.3 Mb), 16′059 (36.1 Mb) and 7′004 (14.2 Mb) SNPs, respectively.

### Simulation

In order to have true (known) haplotypes available, a simulation procedure was performed with R software [22]. Homozygosity levels in the real data were used as a reference to adjust simulation parameters in order to have an in silico created population similar to the real data. The simulation can be summarized in the following steps:

1. The 888 individuals that remained after the quality filters were phased with Beagle v3.3 [11] to obtain a set of basis haplotypes from real data.
2. With these haplotypes a library was created by sampling randomly two sets of 1000 haplotypes.
3. Random haplotypes from the library were allowed to recombine and assigned to the founders of the pedigree. The number of crossing over events followed a Poisson distribution with the parameter λ. In order to adjust the recombination rate to the size covered by the markers of the chip, λ was calculated as the ratio between the distance in bp from first to last SNP of each chromosome and the physical size reported by Groenen [23] (also in bp), multiplied by the average length in cM/100.
4. The founders’ haplotypes were dropped along the real data pedigree, simulating the mating, allowing recombination (same parameters as step 3), but no mutation.
5. At this point all the individuals presented known haplotypes, but for the following analyses we only used the 888 individuals that would have been available in real data analyses with quality checked genotypes. The 888 individuals’ subsets with known haplotypes were saved as the true haplotypes file.
6. The 888 individuals’ subsets haplotype data were transformed to genotype format (0, 1 and 2) and files were saved as the data to be phased with different software.

This simulation process was repeated ten times resulting in ten replicates per chromosome.

### Subsets

In order to analyze the effect of genotyped close relatives in the sample, three subsets were created from the 888 individuals used. The first subset (None-P) comprised 231 individuals whose parents were not genotyped (37 individuals from this group had progeny). The second subset (One-P) grouped 606 individuals with only one parent genotyped. The last subset (Both-P) contained 51 individuals whose parents were both genotyped. The impact of progeny was also analyzed by creating three subsets with increasing amount of progeny. The first subset grouped individuals without progeny, the second subset individuals which had 1 or 2 progeny and the third subset 2 or more progeny.

### Phasing quality analysis

We phased simulated data of 888 individuals for chromosomes 1, 7 and 20 (ten replicates each) with FImpute v2.2 [8] and Beagle (v3.3 and v4.1) [11]. FImpute was applied in two formats, with and without pedigree information. Analyzing the performance of FImpute without pedigree was of interest since this software relies on the shared haplotypes between relatives for phasing. When the pedigree information is not provided, the algorithm scans haplotypes to find parent-offspring pairs. Parameters for both software were left with default settings. The details can be found in the documentation of FImpute and Beagle (v3.3, v4.1). Both software are freely available for academic purposes.

After the datasets had been phased, the comparison with the true haplotypes was done in an R software environment [22] using the Zoo package [24]. All the individuals’ simulated true haplotypes were compared with their respective phased haplotypes created by FImpute and Beagle. Only the heterozygous SNPs were analyzed, as only these are informative when comparing phases.

We calculated for each of the three chromosomes separately *(i)* the percentage of SNPs which were phased equally compared to the true haplotype (Eqp), *(ii)* the number of breakpoints (Bkp), *(iii)* the physical distance between breakpoints (Mb) and *(iv)* the proportion (%) of completely correctly phased individuals. The Eqp parameter was estimated assuming that the lowest value possible was 50%. If a given Eqp value, Eqp_*i*_ was lower than 50% we assumed that the haplotypes being compared were complementary. In this case, the real Eqp value was calculated as 100% minus Eqp_*i*_.

A Bkp was defined as the physical place in the haplotype where a change of phase was detected compared to the true haplotype. If a given allele of a haplotype obtained from the phasing software was different from the true haplotype, we would consider this part of the haplotype was wrongly phased. Starting from this physical position, SNPs up- and downstream in the haplotype were checked in order to identify if the allele at the given locus was associated to the beginning or the end of a switched/inverted segment of the haplotype. For either beginning or end, the intermediate position between the wrongly phased allele and the adjacent (up- or downstream) correct allele was assumed to be the beginning or the end (depending on the situation) of the inverted segment. The percentage of correctly phased individuals was calculated as the proportion of individuals whose average Eqp value was equal to 1, i.e. that had all the SNPs correctly phased.

**Fig. 7:**
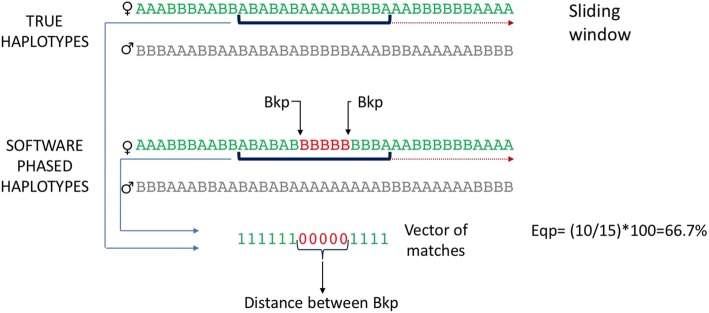
Graphic example of the methodology for calculating Eqp, Bkp and distance between Bkps

Figure 7 shows how the Bkp and the other quality parameters were obtained with a small example. The window of analysis (dark blue) moves from left to right, the vector of matches presents the equally phased (Eqp) SNPs within the window as “1” or unequally phased as “0”. Whenever along the matches vector a sequence of “10” or “01” is observed, this defines a Bkp i.e.*:* a change of phase. The Bkp is also the start or end of a switched segment (in red).

The approach for estimating the quality parameters was performed one chromosome at a time, the details were as follows:

i. The first individual’s haplotypes were read and filtered for heterozygous SNPs.
ii. Individual’s haplotypes (true and phased) were stored as vectors.
iii. A sliding window docked in each haplotype at the first allele. The window covered a fixed length, which was 100, 200 or 400 SNPs.
iv. Within the sliding window, alleles from each haplotype were compared one by one. The matching alleles received a score of “1” otherwise a “0” and these scores were saved in a vector of matches.
v. From the matches vector the Eqp value was calculated as the total number of matches divided by the amount of SNPs scanned (100, 200 or 400) and stored.
vi. If along the vector of matches (Fig. 1) sequences like “10” or “01” were observed these would represent a switch of phase and would be labelled as a Bkp and the respective SNPs’ positions would be stored. The sequence “10” was assumed to be the start of the switched segment while “01” was the end of it.
vii. The value of Eqp and number of Bkp and SNPs position of change of phase were stored.
viii. The sliding window moved one SNP towards the end of the haplotype and steps from *iv* to *vii* were repeated until the haplotype was completely covered.
ix. Median values of Eqp, Bkp and distance of switched segments were calculated for the individual under analysis.
x. The program moved to the next individual and steps from *ii* to *ix* were repeated until all individuals were analyzed.

When the analysis was finished, we had a pair of values of Eqp and Bkp for each position of the sliding window and the positions at which a Bkp was observed along the haplotype for the whole chromosome. Eqp and Bkp values were used to estimate median values over windows per individual. The distances between switched segments were defined as the physical distance between a starting Bkp (10) and a consecutive ending Bkp (01). The distances were calculated through the Bkp positions, stored while observing Bkps, and median values for each individual were obtained. The amount of observations (of Eqp and Bkp) used to calculate the mean/median per individual was determined by the window size (100, 200 or 400) and the number of heterozygous SNPs, thus may differ between individuals.

## Additional file

Additional file 1: Additional file contains **Tables S1-S11** with detailed results for the Figures presented in the main manuscript and in addition results regarding data sets with missing values. (DOCX 131 kb)

## Data Availability

The data that support the findings of this study are available from LOHMANN TIERZUCHT GmbH but restrictions apply to the availability of these data, which were used under license for the current study, and so are not publicly available. Data are however available from the authors upon reasonable request and with permission of LOHMANN TIERZUCHT GmbH.

## Abbreviations

Bkp: Number of incorrectly phased SNPs
Both-P: Subset of individuals with both parents genotyped in the dataset
Eqp: Percentage of SNPs that were phased equally between true and phased haplotypes
HMM: Hidden markov model
LD: Linkage disequilibrium
LHCM: Localized haplotype cluster model
None-P: Subset of individuals with no parent genotyped in the dataset
One-P: Subset of individuals with one parent genotyped in the dataset
OSW: Overlapping sliding window
SNP: Single nucleotide Polymorphism
