# Supplementary material for: Phasing quality assessment in a brown layer population through family- and population-based software
Source: BMC Genet. 2019 Jul 17;20:57. doi: 10.1186/s12863-019-0759-3 (PMC6636125; doi:10.1186/s12863-019-0759-3)
Supplement: Supplementary file 1 — Additional file contains Tables S1-S11 with detailed results for the Figures presented in the main manuscript and in addition results regarding data sets with missing values. (DOCX 131 kb) [file 12863_2019_759_MOESM1_ESM.docx]

Table S1: Results for Chromosome 1: Equally phased and breakpoints average median values for the different window sizes of heterozygous SNPs analyzed, correctly phased percentage of individuals from 100 SNP window and mean and median values of switched segment size in SNPs (absolute values) and bases (percentage relative to the chromosome size).

|  |  | Equally phased | | |  | Breakpoints | | |  | Correctly  phased [%] |  | Switched segment size | | | | |  |  |
| --- | --- | --- | --- | --- | --- | --- | --- | --- | --- | --- | --- | --- | --- | --- | --- | --- | --- | --- |
| Phasing  software | Window | 100 | 200 | 400 |  | 100 | 200 | 400 |  |  |  | SNP | | Percentage of Bases | | | |  |
|  | Subset |  |  |  |  |  |  |  |  |  |  | Mean | Median | | Mean | Median | | |
| FImpute | None-P | 96.22 | 94.87 | 92.96 |  | 1.99 | 3.99 | 7.97 |  | 0.15 |  | 121.29 | 18.09 | | 3.3E-07 | 9.0E-08 | | |
|  | One-P | 99.63 | 99.40 | 99.21 |  | 0.13 | 0.26 | 0.52 |  | 72.92 |  | 86.22 | 63.30 | | 2.9E-07 | 2.2E-07 | | |
|  | Both-P | 99.85 | 99.79 | 99.77 |  | 0.12 | 0.23 | 0.46 |  | 88.04 |  | 25.15 | 11.75 | | 1.0E-07 | 6.0E-08 | | |
|  | Total | 98.81 | 98.30 | 97.66 |  | 0.56 | 1.13 | 2.25 |  | 54.63 |  | 108.01 | 57.42 | | 3.3E-07 | 2.0E-07 | | |
| FImpute  no pedigree | None-P | 97.35 | 95.60 | 92.84 |  | 0.62 | 1.25 | 2.50 |  | 0.21 |  | 191.55 | 17.75 | | 5.3E-07 | 9.5E-08 | | |
|  | One-P | 97.67 | 96.53 | 94.99 |  | 0.88 | 1.77 | 3.54 |  | 8.11 |  | 65.14 | 13.01 | | 2.1E-07 | 6.0E-08 | | |
|  | Both-P | 99.78 | 99.56 | 99.41 |  | 0.06 | 0.12 | 0.24 |  | 78.63 |  | 43.71 | 24.09 | | 2.2E-07 | 1.6E-07 | | |
|  | Total | 97.58 | 96.29 | 94.45 |  | 0.83 | 1.67 | 3.34 |  | 6.16 |  | 102.23 | 17.18 | | 3.1E-07 | 7.5E-08 | | |
| Beagle 3 | None-P | 98.97 | 98.14 | 96.52 |  | 0.11 | 0.22 | 0.44 |  | 11.60 |  | 1742.95 | 1285.82 | | 4.7E-06 | 3.7E-06 | | |
|  | One-P | 99.06 | 98.25 | 96.66 |  | 0.06 | 0.12 | 0.23 |  | 11.70 |  | 1929.92 | 1490.39 | | 5.2E-06 | 4.3E-06 | | |
|  | Both-P | 99.06 | 98.21 | 96.63 |  | 0.05 | 0.10 | 0.20 |  | 11.18 |  | 1965.24 | 1533.07 | | 5.4E-06 | 4.5E-06 | | |
|  | Total | 99.04 | 98.24 | 96.65 |  | 0.07 | 0.14 | 0.28 |  | 12.08 |  | 1911.22 | 1470.92 | | 5.2E-06 | 4.2E-06 | | |
| Beagle 4 | None-P | 99.85 | 99.62 | 99.23 |  | 0.04 | 0.08 | 0.17 |  | 88.45 |  | 1830.57 | 1545.96 | | 4.9E-06 | 4.2E-06 | | |
|  | One-P | 99.97 | 99.83 | 99.56 |  | 0.01 | 0.02 | 0.05 |  | 96.64 |  | 2006.97 | 1803.10 | | 5.4E-06 | 4.9E-06 | | |
|  | Both-P | 99.99 | 99.90 | 99.70 |  | 0.01 | 0.01 | 0.02 |  | 98.82 |  | 2094.93 | 2011.04 | | 5.9E-06 | 5.6E-06 | | |
|  | Total | 99.94 | 99.79 | 99.49 |  | 0.02 | 0.04 | 0.07 |  | 94.93 |  | 1946.97 | 1719.76 | | 5.2E-06 | 4.7E-06 | | |
|  |  |  |  |  |  |  |  |  |  |  |  |  |  | |  |  | | |

Table S2: Results for Chromosome 7: Equally phased and breakpoints average median values for the different window sizes of heterozygous SNPs analyzed, correctly phased percentage of individuals from 100 SNP window and mean and median values of switched segment size in SNPs (absolute values) and bases (percentage relative to the chromosome size).

|  |  | Equally phased | | |  | Breakpoints | | |  | Correctly  phased [%] |  | Switched segment size | | | |  |
| --- | --- | --- | --- | --- | --- | --- | --- | --- | --- | --- | --- | --- | --- | --- | --- | --- |
| Phasing  software | Window | 100 | 200 | 400 |  | 100 | 200 | 400 |  |  |  | SNP | | Percentage of Bases | | |
|  | Subset |  |  |  |  |  |  |  |  |  |  | Mean | Median | Mean | Median | |
| FImpute | None-P | 95.73 | 94.16 | 92.05 |  | 2.15 | 4.32 | 8.64 |  | 4.54 |  | 98.30 | 69.59 | 9.4E-06 | 6.4E-06 | |
|  | One-P | 99.56 | 99.35 | 99.10 |  | 0.12 | 0.24 | 0.48 |  | 75.46 |  | 23.70 | 21.48 | 7.6E-06 | 7.0E-06 | |
|  | Both-P | 99.83 | 99.81 | 99.78 |  | 0.09 | 0.18 | 0.36 |  | 88.82 |  | 5.70 | 4.91 | 3.0E-06 | 2.7E-06 | |
|  | Total | 98.64 | 98.09 | 97.36 |  | 0.59 | 1.18 | 2.36 |  | 57.70 |  | 45.05 | 35.96 | 8.8E-06 | 7.1E-06 | |
| FImpute  no pedigree | None-P | 94.35 | 91.95 | 88.71 |  | 2.62 | 5.30 | 10.64 |  | 10.46 |  | 60.00 | 41.89 | 7.4E-06 | 4.9E-06 | |
|  | One-P | 95.60 | 93.90 | 91.66 |  | 2.13 | 4.31 | 8.63 |  | 25.91 |  | 41.45 | 29.01 | 5.9E-06 | 4.0E-06 | |
|  | Both-P | 99.23 | 98.83 | 98.31 |  | 0.26 | 0.52 | 1.01 |  | 67.65 |  | 42.60 | 37.04 | 1.0E-05 | 9.3E-06 | |
|  | Total | 95.34 | 93.49 | 91.02 |  | 2.23 | 4.50 | 9.03 |  | 22.49 |  | 45.97 | 32.07 | 6.3E-06 | 4.2E-06 | |
| Beagle 3 | None-P | 98.88 | 98.04 | 96.22 |  | 0.11 | 0.22 | 0.44 |  | 24.12 |  | 444.42 | 432.80 | 6.0E-05 | 5.8E-05 | |
|  | One-P | 99.02 | 98.27 | 96.58 |  | 0.06 | 0.12 | 0.23 |  | 27.37 |  | 449.92 | 440.99 | 7.0E-05 | 6.8E-05 | |
|  | Both-P | 99.03 | 98.35 | 96.70 |  | 0.05 | 0.10 | 0.20 |  | 24.90 |  | 439.99 | 435.21 | 7.2E-05 | 7.1E-05 | |
|  | Total | 99.00 | 98.23 | 96.52 |  | 0.07 | 0.14 | 0.28 |  | 26.98 |  | 447.07 | 437.71 | 6.8E-05 | 6.6E-05 | |
| Beagle 4 | None-P | 99.70 | 99.53 | 99.09 |  | 0.04 | 0.08 | 0.16 |  | 76.91 |  | 40.42 | 37.20 | 2.2E-05 | 2.0E-05 | |
|  | One-P | 99.84 | 99.75 | 99.52 |  | 0.01 | 0.02 | 0.05 |  | 85.84 |  | 21.85 | 21.32 | 2.2E-05 | 2.2E-05 | |
|  | Both-P | 99.89 | 99.86 | 99.71 |  | 0.01 | 0.01 | 0.02 |  | 89.41 |  | 1.80 | 1.82 | 5.0E-06 | 5.0E-06 | |
|  | Total | 99.81 | 99.70 | 99.43 |  | 0.02 | 0.04 | 0.07 |  | 84.09 |  | 25.55 | 24.44 | 2.2E-05 | 2.1E-05 | |
|  |  |  |  |  |  |  |  |  |  |  |  |  |  |  |  | |

Table S3: Results for Chromosome 20: Equally phased and breakpoints average median values for the different window sizes of heterozygous SNPs analyzed, correctly phased percentage of individuals from 100 SNP window and mean and median values of switched segment size in SNPs (absolute values) and bases (percentage relative to the chromosome size).

|  |  | Equally phased | | |  | Breakpoints | | |  | Correctly  phased [%] |  | Switched segment size | | | |  |
| --- | --- | --- | --- | --- | --- | --- | --- | --- | --- | --- | --- | --- | --- | --- | --- | --- |
| Phasing  software | Window | 100 | 200 | 400 |  | 100 | 200 | 400 |  |  |  | SNP | | Percentage of Bases | | |
|  | Subset |  |  |  |  |  |  |  |  |  |  | Mean | Median | Mean | Median | |
| FImpute | None-P | 96.03 | 94.47 | 92.32 |  | 1.91 | 3.88 | 7.98 |  | 27.84 |  | 81.52 | 62.54 | 4.1E-05 | 3.3E-07 | |
|  | One-P | 99.57 | 99.40 | 99.20 |  | 0.11 | 0.22 | 0.44 |  | 84.25 |  | 50.16 | 44.68 | 2.5E-05 | 2.3E-07 | |
|  | Both-P | 99.83 | 99.78 | 99.75 |  | 0.07 | 0.16 | 0.33 |  | 93.12 |  | 11.42 | 9.82 | 3.6E-06 | 3.2E-08 | |
|  | Total | 98.75 | 98.24 | 97.57 |  | 0.52 | 1.05 | 2.15 |  | 70.55 |  | 70.89 | 57.91 | 3.5E-05 | 3.0E-07 | |
| FImpute  no pedigree | None-P | 96.17 | 94.45 | 92.20 |  | 1.51 | 3.03 | 6.06 |  | 41.91 |  | 83.80 | 63.17 | 4.3E-05 | 3.4E-07 | |
|  | One-P | 97.31 | 96.11 | 94.52 |  | 1.03 | 2.06 | 4.13 |  | 52.60 |  | 91.44 | 71.79 | 4.6E-05 | 3.8E-07 | |
|  | Both-P | 99.00 | 98.45 | 97.90 |  | 0.26 | 0.53 | 1.05 |  | 72.30 |  | 74.81 | 62.19 | 4.1E-05 | 3.5E-07 | |
|  | Total | 97.10 | 95.79 | 94.08 |  | 1.12 | 2.24 | 4.48 |  | 50.57 |  | 90.36 | 70.61 | 4.5E-05 | 3.7E-07 | |
| Beagle 3 | None-P | 99.18 | 98.49 | 97.09 |  | 0.10 | 0.19 | 0.39 |  | 64.74 |  | 316.55 | 306.15 | 1.6E-04 | 1.6E-06 | |
|  | One-P | 99.33 | 98.66 | 97.41 |  | 0.05 | 0.10 | 0.19 |  | 66.61 |  | 309.11 | 302.72 | 1.7E-04 | 1.7E-06 | |
|  | Both-P | 99.38 | 98.70 | 97.59 |  | 0.04 | 0.07 | 0.14 |  | 69.74 |  | 307.13 | 306.06 | 1.9E-04 | 1.9E-06 | |
|  | Total | 99.30 | 98.63 | 97.35 |  | 0.06 | 0.12 | 0.24 |  | 66.35 |  | 311.58 | 303.97 | 1.7E-04 | 1.7E-06 | |
| Beagle 4 | None-P | 99.73 | 99.52 | 99.15 |  | 0.04 | 0.07 | 0.15 |  | 87.68 |  | 122.67 | 113.62 | 7.0E-05 | 6.7E-07 | |
|  | One-P | 99.85 | 99.72 | 99.47 |  | 0.01 | 0.03 | 0.05 |  | 92.27 |  | 136.08 | 129.12 | 6.3E-05 | 6.0E-07 | |
|  | Both-P | 99.89 | 99.79 | 99.60 |  | 0.01 | 0.01 | 0.03 |  | 93.91 |  | 50.46 | 50.46 | 2.7E-05 | 2.7E-07 | |
|  | Total | 99.83 | 99.68 | 99.41 |  | 0.02 | 0.04 | 0.07 |  | 91.37 |  | 128.57 | 120.96 | 6.4E-05 | 6.1E-07 | |
|  |  |  |  |  |  |  |  |  |  |  |  |  |  |  |  | |

Table S4: Results for Chromosome 1 with missing values: Equally phased and breakpoints average median values for the different window sizes of heterozygous SNPs analyzed, correctly phased percentage of individuals from 100 SNP window and mean and median values of switched segment size in SNPs (absolute values) and bases (percentage relative to the chromosome size).

|  |  | Equally phased | | |  | Breakpoints | | |  | Correctly  phased [%] |  | Switched segment size | | | | |  |  |
| --- | --- | --- | --- | --- | --- | --- | --- | --- | --- | --- | --- | --- | --- | --- | --- | --- | --- | --- |
| Phasing  software | Window | 100 | 200 | 400 |  | 100 | 200 | 400 |  |  |  | SNP | | Percentage of Bases | | | |  |
|  | Subset |  |  |  |  |  |  |  |  |  |  | Mean | Median | | Mean | Median | | |
| FImpute | None-P | 95.99 | 94.53 | 92.49 |  | 2.12 | 4.26 | 8.52 |  | 0.00 |  | 114.12 | 18.06 | | 3.2E-09 | 9.0E-08 | | |
|  | One-P | 99.61 | 99.38 | 99.19 |  | 0.16 | 0.33 | 0.66 |  | 71.66 |  | 64.21 | 42.66 | | 2.1E-09 | 1.5E-07 | | |
|  | Both-P | 99.85 | 99.81 | 99.79 |  | 0.14 | 0.27 | 0.54 |  | 86.54 |  | 10.51 | 4.18 | | 4.0E-10 | 2.0E-08 | | |
|  | Total | 98.74 | 98.21 | 97.55 |  | 0.63 | 1.26 | 2.52 |  | 53.69 |  | 89.72 | 45.59 | | 2.7E-09 | 1.6E-07 | | |
| FImpute  no pedigree | None-P | 97.22 | 95.43 | 92.59 |  | 0.69 | 1.38 | 2.77 |  | 0.21 |  | 175.81 | 16.59 | | 5.0E-09 | 8.5E-08 | | |
|  | One-P | 97.58 | 96.37 | 94.74 |  | 0.90 | 1.81 | 3.62 |  | 6.69 |  | 62.10 | 9.77 | | 2.1E-09 | 5.0E-08 | | |
|  | Both-P | 99.68 | 99.43 | 99.21 |  | 0.09 | 0.18 | 0.36 |  | 68.10 |  | 38.30 | 21.41 | | 2.0E-09 | 1.4E-07 | | |
|  | Total | 97.50 | 96.18 | 94.29 |  | 0.87 | 1.74 | 3.49 |  | 5.10 |  | 92.64 | 12.27 | | 2.8E-09 | 6.0E-08 | | |
| Beagle 3 | None-P | 98.91 | 98.02 | 96.28 |  | 0.12 | 0.23 | 0.47 |  | 8.25 |  | 1436.16 | 993.78 | | 4.0E-08 | 3.1E-06 | | |
|  | One-P | 98.99 | 98.11 | 96.40 |  | 0.07 | 0.13 | 0.26 |  | 8.47 |  | 1670.92 | 1203.17 | | 4.6E-08 | 3.7E-06 | | |
|  | Both-P | 98.96 | 98.05 | 96.21 |  | 0.06 | 0.12 | 0.24 |  | 5.36 |  | 1716.18 | 1297.86 | | 4.8E-08 | 4.0E-06 | | |
|  | Total | 99.02 | 98.19 | 96.54 |  | 0.07 | 0.15 | 0.29 |  | 8.75 |  | 1725.08 | 1242.28 | | 4.7E-08 | 3.7E-06 | | |
| Beagle 4 | None-P | 99.83 | 99.57 | 99.14 |  | 0.05 | 0.11 | 0.21 |  | 86.70 |  | 1423.46 | 1125.97 | | 3.9E-08 | 3.1E-06 | | |
|  | One-P | 99.96 | 99.81 | 99.51 |  | 0.02 | 0.04 | 0.07 |  | 96.30 |  | 1475.79 | 1249.81 | | 4.0E-08 | 3.4E-06 | | |
|  | Both-P | 99.99 | 99.87 | 99.61 |  | 0.01 | 0.02 | 0.03 |  | 99.08 |  | 1457.22 | 1393.19 | | 4.2E-08 | 4.0E-06 | | |
|  | Total | 99.94 | 99.77 | 99.45 |  | 0.02 | 0.05 | 0.10 |  | 94.29 |  | 1448.62 | 1187.80 | | 3.8E-08 | 3.2E-06 | | |
|  |  |  |  |  |  |  |  |  |  |  |  |  |  | |  |  | | |

Table S5: Results for Chromosome 7 with missing values: Equally phased and breakpoints average median values for the different window sizes of heterozygous SNPs analyzed, correctly phased percentage of individuals from 100 SNP window and mean and median values of switched segment size in SNPs (absolute values) and bases (percentage relative to the chromosome size).

|  |  | Equally phased | | |  | Breakpoints | | |  | Correctly  phased [%] |  | Switched segment size | | | |  |
| --- | --- | --- | --- | --- | --- | --- | --- | --- | --- | --- | --- | --- | --- | --- | --- | --- |
| Phasing  software | Window | 100 | 200 | 400 |  | 100 | 200 | 400 |  |  |  | SNP | | Percentage of Bases | | |
|  | Subset |  |  |  |  |  |  |  |  |  |  | Mean | Median | Mean | Median | |
| FImpute | None-P | 95.80 | 94.26 | 92.09 |  | 2.08 | 4.14 | 8.13 |  | 6.32 |  | 116.21 | 71.64 | 8.5E-06 | 5.9E-06 | |
|  | One-P | 99.56 | 99.36 | 99.09 |  | 0.14 | 0.27 | 0.54 |  | 75.18 |  | 51.03 | 44.57 | 4.1E-06 | 3.6E-06 | |
|  | Both-P | 99.81 | 99.76 | 99.69 |  | 0.09 | 0.17 | 0.34 |  | 88.24 |  | 31.76 | 29.83 | 3.3E-06 | 3.2E-06 | |
|  | Total | 98.76 | 98.29 | 97.62 |  | 0.55 | 1.09 | 2.14 |  | 57.87 |  | 80.98 | 61.17 | 6.3E-06 | 5.0E-06 | |
| FImpute  no pedigree | None-P | 94.09 | 91.69 | 88.38 |  | 2.89 | 5.83 | 11.71 |  | 10.93 |  | 77.31 | 42.49 | 6.3E-06 | 4.0E-06 | |
|  | One-P | 95.32 | 93.54 | 91.16 |  | 2.36 | 4.76 | 9.53 |  | 25.94 |  | 58.37 | 36.04 | 4.7E-06 | 3.2E-06 | |
|  | Both-P | 99.27 | 98.88 | 98.42 |  | 0.28 | 0.56 | 1.15 |  | 68.37 |  | 70.14 | 61.71 | 6.9E-06 | 6.2E-06 | |
|  | Total | 95.01 | 93.12 | 90.57 |  | 2.53 | 5.10 | 10.17 |  | 22.64 |  | 63.50 | 37.63 | 5.0E-06 | 3.3E-06 | |
| Beagle 3 | None-P | 98.87 | 98.00 | 96.05 |  | 0.11 | 0.21 | 0.44 |  | 23.71 |  | 705.19 | 669.14 | 5.1E-05 | 4.9E-05 | |
|  | One-P | 98.97 | 98.15 | 96.34 |  | 0.07 | 0.13 | 0.27 |  | 25.60 |  | 795.90 | 771.93 | 5.8E-05 | 5.7E-05 | |
|  | Both-P | 98.95 | 98.12 | 96.23 |  | 0.06 | 0.11 | 0.23 |  | 22.61 |  | 976.66 | 958.35 | 6.8E-05 | 6.7E-05 | |
|  | Total | 99.04 | 98.29 | 96.60 |  | 0.07 | 0.15 | 0.30 |  | 25.53 |  | 743.83 | 719.83 | 5.5E-05 | 5.4E-05 | |
| Beagle 4 | None-P | 99.72 | 99.57 | 99.17 |  | 0.04 | 0.08 | 0.17 |  | 78.90 |  | 198.85 | 183.21 | 1.5E-05 | 1.4E-05 | |
|  | One-P | 99.83 | 99.75 | 99.51 |  | 0.02 | 0.04 | 0.07 |  | 85.28 |  | 194.31 | 172.54 | 1.5E-05 | 1.4E-05 | |
|  | Both-P | 99.89 | 99.82 | 99.63 |  | 0.01 | 0.02 | 0.04 |  | 89.93 |  | 131.86 | 129.52 | 1.6E-05 | 1.6E-05 | |
|  | Total | 99.81 | 99.70 | 99.42 |  | 0.03 | 0.05 | 0.11 |  | 84.18 |  | 177.35 | 157.51 | 1.4E-05 | 1.3E-05 | |
|  |  |  |  |  |  |  |  |  |  |  |  |  |  |  |  | |

Table S6: Results for Chromosome 20 with missing values: Equally phased and breakpoints average median values for the different window sizes of heterozygous SNPs analyzed, correctly phased percentage of individuals from 100 SNP window and mean and median values of switched segment size in SNPs (absolute values) and bases (percentage relative to the chromosome size).

|  |  | Equally phased | | |  | Breakpoints | | |  | Correctly  phased [%] |  | Switched segment size | | | |  |
| --- | --- | --- | --- | --- | --- | --- | --- | --- | --- | --- | --- | --- | --- | --- | --- | --- |
| Phasing  software | Window | 100 | 200 | 400 |  | 100 | 200 | 400 |  |  |  | SNP | | Percentage of Bases | | |
|  | Subset |  |  |  |  |  |  |  |  |  |  | Mean | Median | Mean | Median | |
| FImpute | None-P | 95.96 | 94.45 | 92.45 |  | 1.85 | 3.77 | 7.81 |  | 20.03 |  | 95.72 | 79.24 | 4.5E-05 | 3.8E-05 | |
|  | One-P | 99.58 | 99.40 | 99.20 |  | 0.12 | 0.23 | 0.45 |  | 83.46 |  | 46.33 | 41.36 | 2.4E-05 | 2.1E-05 | |
|  | Both-P | 99.89 | 99.87 | 99.87 |  | 0.07 | 0.15 | 0.32 |  | 94.38 |  | 5.64 | 4.16 | 3.2E-06 | 2.8E-06 | |
|  | Total | 98.70 | 98.19 | 97.60 |  | 0.51 | 1.01 | 2.05 |  | 68.12 |  | 65.35 | 52.55 | 3.2E-05 | 2.7E-05 | |
| FImpute  no pedigree | None-P | 95.94 | 94.23 | 91.97 |  | 1.70 | 3.41 | 6.81 |  | 40.45 |  | 85.29 | 67.76 | 4.2E-05 | 3.3E-05 | |
|  | One-P | 97.15 | 95.88 | 94.17 |  | 1.11 | 2.23 | 4.50 |  | 49.74 |  | 89.97 | 71.44 | 4.6E-05 | 3.8E-05 | |
|  | Both-P | 98.84 | 98.27 | 97.52 |  | 0.34 | 0.66 | 1.29 |  | 69.67 |  | 80.35 | 54.18 | 4.1E-05 | 3.1E-05 | |
|  | Total | 96.78 | 95.46 | 93.70 |  | 1.36 | 2.70 | 5.43 |  | 48.12 |  | 80.60 | 62.78 | 4.0E-05 | 3.2E-05 | |
| Beagle 3 | None-P | 99.18 | 98.48 | 97.19 |  | 0.10 | 0.18 | 0.36 |  | 61.68 |  | 289.85 | 278.48 | 1.4E-04 | 1.3E-04 | |
|  | One-P | 99.31 | 98.64 | 97.36 |  | 0.06 | 0.11 | 0.22 |  | 65.36 |  | 290.21 | 283.65 | 1.6E-04 | 1.6E-04 | |
|  | Both-P | 99.44 | 98.87 | 97.83 |  | 0.04 | 0.08 | 0.15 |  | 65.62 |  | 290.32 | 283.76 | 1.5E-04 | 1.5E-04 | |
|  | Total | 99.31 | 98.65 | 97.38 |  | 0.07 | 0.13 | 0.25 |  | 64.85 |  | 236.06 | 227.47 | 1.3E-04 | 1.3E-04 | |
| Beagle 4 | None-P | 99.73 | 99.54 | 99.21 |  | 0.04 | 0.07 | 0.14 |  | 87.73 |  | 124.72 | 118.88 | 7.0E-05 | 6.6E-05 | |
|  | One-P | 99.84 | 99.70 | 99.44 |  | 0.02 | 0.04 | 0.09 |  | 91.82 |  | 81.61 | 73.69 | 4.3E-05 | 4.0E-05 | |
|  | Both-P | 99.90 | 99.80 | 99.61 |  | 0.01 | 0.02 | 0.04 |  | 94.25 |  | 29.57 | 29.58 | 1.6E-05 | 1.6E-05 | |
|  | Total | 99.83 | 99.69 | 99.45 |  | 0.03 | 0.05 | 0.10 |  | 91.09 |  | 77.11 | 69.70 | 4.4E-05 | 4.1E-05 | |
|  |  |  |  |  |  |  |  |  |  |  |  |  |  |  |  | |

Table S7: Results for Chromosome 1,7 and 20: Breakpoints average median values relative to each window size.

| Chromosome | | 1 | | |  | 7 | | |  | 20 | | |
| --- | --- | --- | --- | --- | --- | --- | --- | --- | --- | --- | --- | --- |
| Phasing  software | Window | 100 | 200 | 400 |  | 100 | 200 | 400 |  | 100 | 200 | 400 |
|  |  |  |  |  |  |  |  |  |  |  |  |  |
| FImpute | None-P | 0,020 | 0,020 | 0,020 |  | 0,021 | 0,022 | 0,022 |  | 0,019 | 0,019 | 0,020 |
|  | One-P | 0,001 | 0,001 | 0,001 |  | 0,001 | 0,001 | 0,001 |  | 0,001 | 0,001 | 0,001 |
|  | Both-P | 0,001 | 0,001 | 0,001 |  | 0,001 | 0,001 | 0,001 |  | 0,001 | 0,001 | 0,001 |
|  | Total | 0,006 | 0,006 | 0,006 |  | 0,006 | 0,006 | 0,006 |  | 0,005 | 0,005 | 0,005 |
| Fimpute  no pedigree | None-P | 0,006 | 0,006 | 0,006 |  | 0,026 | 0,026 | 0,027 |  | 0,015 | 0,015 | 0,015 |
|  | One-P | 0,009 | 0,009 | 0,009 |  | 0,021 | 0,022 | 0,022 |  | 0,010 | 0,010 | 0,010 |
|  | Both-P | 0,001 | 0,001 | 0,001 |  | 0,003 | 0,003 | 0,003 |  | 0,003 | 0,003 | 0,003 |
|  | Total | 0,008 | 0,008 | 0,008 |  | 0,022 | 0,022 | 0,023 |  | 0,011 | 0,011 | 0,011 |
| Beagle 3 | None-P | 0,001 | 0,001 | 0,001 |  | 0,001 | 0,001 | 0,001 |  | 0,001 | 0,001 | 0,001 |
|  | One-P | 0,001 | 0,001 | 0,001 |  | 0,001 | 0,001 | 0,001 |  | 0,000 | 0,000 | 0,000 |
|  | Both-P | 0,000 | 0,001 | 0,001 |  | 0,000 | 0,000 | 0,000 |  | 0,000 | 0,000 | 0,000 |
|  | Total | 0,001 | 0,001 | 0,001 |  | 0,001 | 0,001 | 0,001 |  | 0,001 | 0,001 | 0,001 |
| Beagle 4 | None-P | 0,000 | 0,000 | 0,000 |  | 0,000 | 0,000 | 0,000 |  | 0,000 | 0,000 | 0,000 |
|  | One-P | 0,000 | 0,000 | 0,000 |  | 0,000 | 0,000 | 0,000 |  | 0,000 | 0,000 | 0,000 |
|  | Both-P | 0,000 | 0,000 | 0,000 |  | 0,000 | 0,000 | 0,000 |  | 0,000 | 0,000 | 0,000 |
|  | Total | 0,000 | 0,000 | 0,000 |  | 0,000 | 0,000 | 0,000 |  | 0,000 | 0,000 | 0,000 |

Table S8: Equally phased and breakpoints average median values for progeny groups.

|  |  |  |  | Equally phased | | |  | Breakpoints | | |
| --- | --- | --- | --- | --- | --- | --- | --- | --- | --- | --- |
| Chr. | Software | Progeny | Window | 100 | 200 | 400 |  | 100 | 200 | 400 |
| 1 | Fimpute | 0 |  | 1,000 | 1,000 | 0,998 |  | 0,004 | 0,042 | 0,276 |
|  |  | 1 or 2 |  | 1,000 | 1,000 | 0,999 |  | 0,000 | 0,004 | 0,153 |
|  |  | >2 |  | 1,000 | 1,000 | 0,999 |  | 0,000 | 0,008 | 0,067 |
|  | FImpute np | 0 |  | 1,000 | 1,000 | 0,998 |  | 0,001 | 0,022 | 0,160 |
|  |  | 1 or 2 |  | 1,000 | 0,999 | 0,991 |  | 0,004 | 0,086 | 0,494 |
|  |  | >2 |  | 1,000 | 0,999 | 0,993 |  | 0,002 | 0,069 | 0,465 |
|  | Beagle 3 | 0 |  | 1,000 | 1,000 | 1,000 |  | 0,000 | 0,001 | 0,005 |
|  |  | 1 or 2 |  | 1,000 | 1,000 | 1,000 |  | 0,000 | 0,000 | 0,000 |
|  |  | >2 |  | 1,000 | 1,000 | 1,000 |  | 0,000 | 0,000 | 0,000 |
|  | Beagle 4 | 0 |  | 1,000 | 1,000 | 1,000 |  | 0,000 | 0,001 | 0,004 |
|  |  | 1 or 2 |  | 1,000 | 1,000 | 1,000 |  | 0,000 | 0,000 | 0,000 |
|  |  | >2 |  | 1,000 | 1,000 | 1,000 |  | 0,000 | 0,000 | 0,000 |
| 7 | Fimpute | 0 |  | 0,999 | 0,997 | 0,991 |  | 0,040 | 0,191 | 0,849 |
|  |  | 1 or 2 |  | 1,000 | 0,998 | 0,992 |  | 0,018 | 0,133 | 0,730 |
|  |  | >2 |  | 1,000 | 0,999 | 0,996 |  | 0,004 | 0,037 | 0,188 |
|  | FImpute np | 0 |  | 0,995 | 0,979 | 0,944 |  | 0,204 | 0,983 | 3,920 |
|  |  | 1 or 2 |  | 0,998 | 0,986 | 0,957 |  | 0,116 | 0,616 | 2,766 |
|  |  | >2 |  | 0,999 | 0,993 | 0,982 |  | 0,055 | 0,369 | 1,535 |
|  | Beagle 3 | 0 |  | 1,000 | 1,000 | 0,999 |  | 0,001 | 0,003 | 0,027 |
|  |  | 1 or 2 |  | 1,000 | 1,000 | 1,000 |  | 0,000 | 0,000 | 0,006 |
|  |  | >2 |  | 1,000 | 1,000 | 1,000 |  | 0,000 | 0,000 | 0,008 |
|  | Beagle 4 | 0 |  | 1,000 | 1,000 | 1,000 |  | 0,000 | 0,002 | 0,006 |
|  |  | 1 or 2 |  | 1,000 | 1,000 | 1,000 |  | 0,000 | 0,000 | 0,000 |
|  |  | >2 |  | 1,000 | 1,000 | 1,000 |  | 0,000 | 0,000 | 0,000 |
| 20 | Fimpute | 0 |  | 0,998 | 0,994 | 0,986 |  | 0,100 | 0,423 | 1,452 |
|  |  | 1 or 2 |  | 1,000 | 0,998 | 0,988 |  | 0,014 | 0,132 | 0,822 |
|  |  | >2 |  | 1,000 | 0,998 | 0,996 |  | 0,012 | 0,094 | 0,393 |
|  | FImpute np | 0 |  | 0,994 | 0,981 | 0,957 |  | 0,216 | 0,885 | 3,063 |
|  |  | 1 or 2 |  | 0,996 | 0,992 | 0,980 |  | 0,186 | 0,461 | 1,579 |
|  |  | >2 |  | 0,998 | 0,994 | 0,987 |  | 0,025 | 0,165 | 0,633 |
|  | Beagle 3 | 0 |  | 1,000 | 0,999 | 0,993 |  | 0,002 | 0,014 | 0,096 |
|  |  | 1 or 2 |  | 1,000 | 0,999 | 0,996 |  | 0,004 | 0,018 | 0,145 |
|  |  | >2 |  | 1,000 | 0,999 | 0,994 |  | 0,000 | 0,010 | 0,045 |
|  | Beagle 4 | 0 |  | 1,000 | 1,000 | 0,998 |  | 0,001 | 0,004 | 0,030 |
|  |  | 1 or 2 |  | 1,000 | 1,000 | 0,999 |  | 0,002 | 0,006 | 0,012 |
|  |  | >2 |  | 1,000 | 1,000 | 1,000 |  | 0,000 | 0,000 | 0,002 |

Table S9: Results for chromosome 1: Coefficient of variation and standard error across replicates for mean values of equally phased and breakpoints, mean and median values of switched segments size in SNPs and Bp units.

|  |  |  | Equally phased | | | Breakpoints | | | Switched segment size | | | |
| --- | --- | --- | --- | --- | --- | --- | --- | --- | --- | --- | --- | --- |
|  |  |  |  |  |  |  |  |  | SNP | | Bp | |
| Software | Subset | Parameter | 100 | 200 | 300 | 100 | 200 | 300 | Mean | Median | Mean | Median |
| FImpute  no  pedigree | None-P | CV | 0,002 | 0,002 | 0,004 | 0,135 | 0,136 | 0,137 | 0,112 | 0,367 | 0,105 | 0,260 |
|  |  | Std. Err. | 0,000 | 0,001 | 0,001 | 0,027 | 0,054 | 0,109 | 6,801 | 2,062 | 69716,069 | 30644,546 |
|  | One-P | CV | 0,002 | 0,002 | 0,003 | 0,079 | 0,078 | 0,077 | 0,090 | 0,430 | 0,062 | 0,303 |
|  |  | Std. Err. | 0,000 | 0,001 | 0,001 | 0,022 | 0,044 | 0,086 | 1,848 | 1,770 | 16602,164 | 23392,232 |
|  | Both | CV | 0,001 | 0,001 | 0,002 | 0,238 | 0,238 | 0,239 | 0,287 | 0,316 | 0,180 | 0,246 |
|  |  | Std. Err. | 0,000 | 0,000 | 0,000 | 0,004 | 0,009 | 0,018 | 3,931 | 2,385 | 49882,131 | 49227,322 |
| FImpute | None-P | CV | 0,004 | 0,005 | 0,006 | 0,135 | 0,134 | 0,133 | 0,099 | 0,209 | 0,088 | 0,148 |
|  |  | Std. Err. | 0,001 | 0,001 | 0,002 | 0,085 | 0,169 | 0,334 | 3,780 | 1,196 | 36364,708 | 16406,377 |
|  | One-P | CV | 0,000 | 0,000 | 0,001 | 0,099 | 0,101 | 0,104 | 0,294 | 0,378 | 0,226 | 0,271 |
|  |  | Std. Err. | 0,000 | 0,000 | 0,000 | 0,004 | 0,008 | 0,017 | 8,035 | 7,563 | 81204,140 | 74030,555 |
|  | Both | CV | 0,001 | 0,001 | 0,001 | 0,282 | 0,281 | 0,292 | 1,680 | 1,562 | 1,382 | 1,039 |
|  |  | Std. Err. | 0,000 | 0,000 | 0,000 | 0,010 | 0,021 | 0,043 | 13,916 | 6,067 | 184145,248 | 82712,409 |
| Beagle 3 | None-P | CV | 0,001 | 0,001 | 0,002 | 0,084 | 0,084 | 0,087 | 0,102 | 0,138 | 0,075 | 0,100 |
|  |  | Std. Err. | 0,000 | 0,000 | 0,001 | 0,003 | 0,006 | 0,012 | 56,420 | 56,097 | 445487,970 | 469619,378 |
|  | One-P | CV | 0,001 | 0,002 | 0,003 | 0,072 | 0,074 | 0,075 | 0,093 | 0,124 | 0,068 | 0,083 |
|  |  | Std. Err. | 0,000 | 0,001 | 0,001 | 0,001 | 0,003 | 0,006 | 56,651 | 58,460 | 451685,542 | 451284,273 |
|  | Both | CV | 0,001 | 0,002 | 0,003 | 0,062 | 0,063 | 0,062 | 0,128 | 0,165 | 0,112 | 0,132 |
|  |  | Std. Err. | 0,000 | 0,001 | 0,001 | 0,001 | 0,002 | 0,004 | 79,411 | 79,954 | 767558,082 | 748251,331 |
| Beagle 4 | None-P | CV | 0,000 | 0,001 | 0,001 | 0,179 | 0,180 | 0,183 | 0,165 | 0,199 | 0,137 | 0,166 |
|  |  | Std. Err. | 0,000 | 0,000 | 0,000 | 0,002 | 0,005 | 0,010 | 95,822 | 97,215 | 836496,928 | 865191,641 |
|  | One-P | CV | 0,000 | 0,000 | 0,000 | 0,091 | 0,088 | 0,085 | 0,119 | 0,129 | 0,114 | 0,118 |
|  |  | Std. Err. | 0,000 | 0,000 | 0,000 | 0,000 | 0,001 | 0,001 | 75,684 | 73,506 | 772919,573 | 727469,089 |
|  | Both | CV | 0,000 | 0,000 | 0,001 | 0,195 | 0,197 | 0,197 | 0,222 | 0,234 | 0,183 | 0,197 |
|  |  | Std. Err. | 0,000 | 0,000 | 0,000 | 0,000 | 0,001 | 0,001 | 148,367 | 148,701 | 1353329,348 | 1390197,990 |

Table S10: Results for chromosome 7: Coefficient of variation and standard error across replicates for mean values of equally phased and breakpoints, mean and median values of switched segments size in SNPs and Bp units.

|  |  |  | Equally phased | | | Breakpoints | | | Switched segment size | | | |
| --- | --- | --- | --- | --- | --- | --- | --- | --- | --- | --- | --- | --- |
|  |  |  |  |  |  |  |  |  | SNP | | Bp | |
| Software | Subset | Parameter | 100 | 200 | 300 | 100 | 200 | 300 | Mean | Median | Mean | Median |
| FImpute  no  pedigree | None-P | CV | 0,005 | 0,006 | 0,009 | 0,164 | 0,165 | 0,174 | 0,178 | 0,312 | 0,165 | 0,247 |
|  |  | Std. Err. | 0,001 | 0,002 | 0,003 | 0,112 | 0,226 | 0,475 | 6,902 | 7,379 | 65911,918 | 66185,024 |
|  | One-P | CV | 0,001 | 0,001 | 0,001 | 0,222 | 0,220 | 0,217 | 0,175 | 0,190 | 0,181 | 0,198 |
|  |  | Std. Err. | 0,000 | 0,000 | 0,000 | 0,008 | 0,017 | 0,033 | 3,276 | 3,116 | 43437,939 | 43237,848 |
|  | Both | CV | 0,001 | 0,001 | 0,001 | 0,405 | 0,400 | 0,402 | 1,270 | 1,599 | 1,165 | 1,349 |
|  |  | Std. Err. | 0,000 | 0,000 | 0,000 | 0,012 | 0,023 | 0,045 | 6,669 | 6,687 | 115686,732 | 119069,083 |
| FImpute | None-P | CV | 0,005 | 0,007 | 0,009 | 0,109 | 0,109 | 0,108 | 0,366 | 0,604 | 0,275 | 0,407 |
|  |  | Std. Err. | 0,001 | 0,002 | 0,002 | 0,090 | 0,183 | 0,365 | 10,302 | 9,731 | 87519,437 | 84370,153 |
|  | One-P | CV | 0,003 | 0,005 | 0,007 | 0,077 | 0,079 | 0,078 | 0,245 | 0,374 | 0,194 | 0,258 |
|  |  | Std. Err. | 0,001 | 0,001 | 0,002 | 0,052 | 0,108 | 0,212 | 4,787 | 4,365 | 45130,458 | 40242,186 |
|  | Both | CV | 0,002 | 0,004 | 0,006 | 0,375 | 0,374 | 0,385 | 1,153 | 1,262 | 0,762 | 0,844 |
|  |  | Std. Err. | 0,001 | 0,001 | 0,002 | 0,030 | 0,061 | 0,123 | 32,323 | 30,047 | 267210,679 | 260201,078 |
| Beagle 3 | None-P | CV | 0,001 | 0,002 | 0,004 | 0,145 | 0,149 | 0,145 | 0,194 | 0,205 | 0,139 | 0,146 |
|  |  | Std. Err. | 0,000 | 0,001 | 0,001 | 0,005 | 0,010 | 0,020 | 47,876 | 48,767 | 364059,077 | 370836,449 |
|  | One-P | CV | 0,001 | 0,003 | 0,005 | 0,142 | 0,139 | 0,137 | 0,087 | 0,093 | 0,056 | 0,060 |
|  |  | Std. Err. | 0,000 | 0,001 | 0,001 | 0,003 | 0,005 | 0,010 | 24,697 | 25,673 | 170036,475 | 176879,387 |
|  | Both | CV | 0,002 | 0,004 | 0,007 | 0,208 | 0,211 | 0,213 | 0,215 | 0,214 | 0,106 | 0,103 |
|  |  | Std. Err. | 0,001 | 0,001 | 0,002 | 0,003 | 0,006 | 0,013 | 62,715 | 62,086 | 330459,223 | 318634,292 |
| Beagle 4 | None-P | CV | 0,001 | 0,001 | 0,002 | 0,357 | 0,353 | 0,356 | 0,385 | 0,448 | 0,329 | 0,373 |
|  |  | Std. Err. | 0,000 | 0,000 | 0,001 | 0,004 | 0,009 | 0,018 | 34,134 | 35,626 | 325935,125 | 340429,197 |
|  | One-P | CV | 0,000 | 0,001 | 0,001 | 0,209 | 0,220 | 0,231 | 0,322 | 0,360 | 0,267 | 0,279 |
|  |  | Std. Err. | 0,000 | 0,000 | 0,000 | 0,001 | 0,002 | 0,003 | 27,059 | 28,975 | 262037,822 | 265479,793 |
|  | Both | CV | 0,001 | 0,001 | 0,001 | 0,523 | 0,534 | 0,577 | 0,943 | 0,943 | 0,937 | 0,937 |
|  |  | Std. Err. | 0,000 | 0,000 | 0,000 | 0,001 | 0,002 | 0,004 | 4,986 | 4,986 | 150326,566 | 150326,566 |

Table S11: Results for chromosome 20: Coefficient of variation and standard error across replicates for mean values of equally phased and breakpoints, mean and median values of switched segments size in SNPs and Bp units.

|  |  |  | Equally phased | | | Breakpoints | | | Switched segment size | | | |
| --- | --- | --- | --- | --- | --- | --- | --- | --- | --- | --- | --- | --- |
|  |  |  |  |  |  |  |  |  | SNP | | Bp | |
| Software | Subset | Parameter | 100 | 200 | 300 | 100 | 200 | 300 | Mean | Median | Mean | Median |
| FImpute  no  pedigree | None-P | CV | 0,008 | 0,010 | 0,014 | 0,285 | 0,290 | 0,291 | 0,239 | 0,354 | 0,179 | 0,241 |
|  |  | Std. Err. | 0,003 | 0,003 | 0,004 | 0,172 | 0,357 | 0,736 | 6,161 | 7,025 | 44752,686 | 48993,350 |
|  | One-P | CV | 0,001 | 0,001 | 0,002 | 0,324 | 0,334 | 0,346 | 0,280 | 0,308 | 0,268 | 0,286 |
|  |  | Std. Err. | 0,000 | 0,000 | 0,000 | 0,011 | 0,023 | 0,048 | 4,473 | 4,394 | 41671,789 | 40750,413 |
|  | Both | CV | 0,001 | 0,002 | 0,002 | 0,643 | 0,733 | 0,665 | 0,620 | 0,821 | 0,482 | 0,609 |
|  |  | Std. Err. | 0,000 | 0,001 | 0,001 | 0,015 | 0,037 | 0,070 | 2,177 | 2,367 | 9672,821 | 10351,257 |
| FImpute | None-P | CV | 0,007 | 0,009 | 0,011 | 0,217 | 0,221 | 0,225 | 0,223 | 0,305 | 0,203 | 0,264 |
|  |  | Std. Err. | 0,002 | 0,003 | 0,003 | 0,103 | 0,211 | 0,431 | 5,972 | 6,194 | 53411,658 | 54878,505 |
|  | One-P | CV | 0,004 | 0,005 | 0,006 | 0,204 | 0,210 | 0,220 | 0,261 | 0,331 | 0,221 | 0,277 |
|  |  | Std. Err. | 0,001 | 0,001 | 0,002 | 0,066 | 0,136 | 0,287 | 7,591 | 7,565 | 61915,963 | 64015,980 |
|  | Both | CV | 0,004 | 0,007 | 0,009 | 0,726 | 0,732 | 0,733 | 0,656 | 0,789 | 0,420 | 0,520 |
|  |  | Std. Err. | 0,001 | 0,002 | 0,003 | 0,061 | 0,122 | 0,242 | 14,653 | 14,673 | 103859,762 | 111174,767 |
| Beagle 3 | None-P | CV | 0,001 | 0,003 | 0,006 | 0,166 | 0,246 | 0,258 | 0,346 | 0,363 | 0,221 | 0,232 |
|  |  | Std. Err. | 0,000 | 0,001 | 0,002 | 0,005 | 0,015 | 0,032 | 34,842 | 35,323 | 217331,942 | 221532,186 |
|  | One-P | CV | 0,002 | 0,003 | 0,006 | 0,231 | 0,237 | 0,258 | 0,223 | 0,234 | 0,168 | 0,177 |
|  |  | Std. Err. | 0,001 | 0,001 | 0,002 | 0,004 | 0,007 | 0,016 | 21,280 | 21,785 | 171629,999 | 177436,560 |
|  | Both | CV | 0,002 | 0,004 | 0,011 | 0,291 | 0,282 | 0,357 | 0,674 | 0,679 | 0,385 | 0,389 |
|  |  | Std. Err. | 0,001 | 0,001 | 0,003 | 0,003 | 0,007 | 0,016 | 67,239 | 67,510 | 436216,209 | 439319,204 |
| Beagle 4 | None-P | CV | 0,001 | 0,001 | 0,002 | 0,258 | 0,338 | 0,378 | 0,333 | 0,349 | 0,303 | 0,315 |
|  |  | Std. Err. | 0,000 | 0,000 | 0,001 | 0,003 | 0,008 | 0,018 | 12,825 | 12,446 | 128509,122 | 127296,181 |
|  | One-P | CV | 0,001 | 0,001 | 0,002 | 0,350 | 0,376 | 0,391 | 0,343 | 0,372 | 0,298 | 0,313 |
|  |  | Std. Err. | 0,000 | 0,000 | 0,001 | 0,001 | 0,003 | 0,006 | 15,038 | 15,532 | 117619,938 | 118762,941 |
|  | Both | CV | 0,001 | 0,002 | 0,004 | 0,524 | 0,540 | 0,624 | 1,169 | 1,169 | 0,911 | 0,911 |
|  |  | Std. Err. | 0,000 | 0,001 | 0,001 | 0,001 | 0,002 | 0,005 | 15,550 | 15,550 | 147672,061 | 147672,061 |
